# Towards a unification of the 2 meanings of “epigenetics”

**DOI:** 10.1371/journal.pbio.3001944

**Published:** 2022-12-27

**Authors:** Sui Huang

**Affiliations:** Institute for Systems Biology, Seattle, Washington, United States of America

## Abstract

The notion of epigenetic “marks” used by molecular biologists is conceptually disconnected from the idea of Waddington’s epigenetic “landscape” that is used by systems biologists and biophysicists. Recent advances suggest that these 2 distinct schools of thought could be united.

Unbeknownst to many, the same term “epigenetics” is used in 2 semantically non-overlapping ways by 2 schools of thought that rarely interact. I refer to them here as molecular epigenetics and systems epigenetics. These 2 schools of epigenetics are largely agnostic of each other, and only an assessment from the vantage points of both schools simultaneously can unify them in a logically precise manner within 1 framework.

Molecular epigenetics pertains to the meaning of epigenetics that is prevalent today and has been adopted by molecular biologists to indicate DNA methylation and those posttranslational modifications of histone proteins that act as epigenetic “marks” that control the activity of a gene locus (Fig 1, left-hand side). These marks are thought to persist, and hence confer memory of a gene’s activity state [1,2]. Molecular epigenetics is also concerned with packaging genomic DNA into compact chromatin [1]. Epigenetic marks recruit proteins that remodel chromatin, shifting it between open and closed configurations. Since gene loci within closed chromatin structures are inaccessible to transcription factors, selective (un)packing of chromatin regions is thought to add a layer of gene expression control that supersedes the regulation by transcription factors.

**Fig 1:**
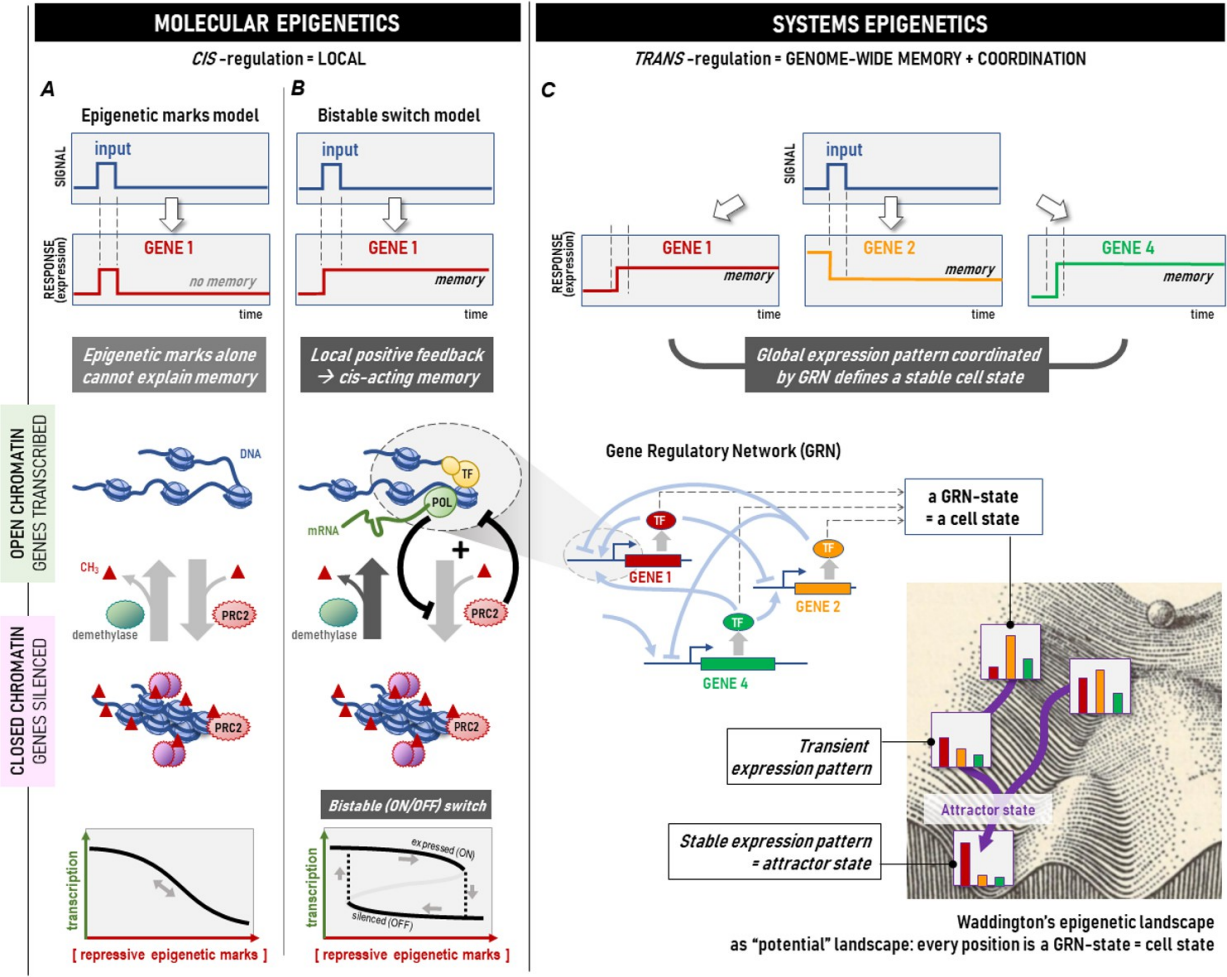
Two schools of “epigenetics”: molecular epigenetics versus systems epigenetics. New studies suggest that local epigenetic regulation by adding or removing epigenetic marks, here exemplified by methylation (red triangle; panel A), is converted to a local bistable (ON-OFF) switch (the *cis*-acting memory; panel B), affording memory but only for 1 gene locus. To produce stable gene expression patterns (shown here as genes 1, 2, and 4), *trans*-regulation, embodied by the GRN (panel C) is required. The dynamics of the GRN as a whole are epitomized by Waddington’s “epigenetic landscape” [3], in which valleys represent stable “attractor states”. GRN, gene regulatory network; POL, polymerase; PRC2, Polycomb complex PRC2; TF, transcription factor.

The paradigm of molecular epigenetics harbors a series of logical inconsistencies [3–5]. First, if modifier enzymes (which add, erase, and read epigenetic marks and cannot recognize specific DNA sequence motifs) control the transcriptional regulation by transcription factors, who controls the controller? Second, if transcription factors must recruit these sequence-agnostic enzymes to open the closed chromatin to access their specific response motifs, how can these transcription factors bind to their inaccessible target sequences? To solve this conundrum, “pioneer factors” capable of binding specific sequences in closed chromatin were once proposed. But if they can access binding sites irrespective of chromatin conformation, they would negate an absolutely necessary role of chromatin in gene regulation. Indeed, the concept of pioneer factors could not be generalized [6]. Third, the ubiquitous writers and erasers of epigenetic marks neutralize each other. How can lasting memory be generated by marks that are dynamic and reversible? Fourth, is chromatin opening a cause or consequence of transcription? While removing repressive methylation marks activates genes, transcriptional activation can also precede demethylation [7]. Such chicken-and-egg schemes are hallmarks of an autoregulatory circuitry.

By contrast, systems epigenetics refers to an older meaning of epigenetics that predates molecular biology and goes back to Conrad Waddington’s “epigenetic landscape.” Used today by systems biologists who seek to compute the actual topography of the landscape [3], it denotes the generation and maintenance of stable patterns of gene expression that underlie a persistent cell phenotype, such as a cell type. A “pattern” requires the expression of each gene to be tightly coordinated with that of other genes, which is achieved by a network of regulatory interactions exerted by transcription factors, each modulating the activity of a specific set of gene loci. They collectively establish the genome-wide gene regulatory network (GRN). Memory arises because mutual regulation of genes in the GRN can “lock-in” their respective expression states, producing a stable expression pattern: the “attractor states” epitomized by the valleys in Waddington’s landscape [3] (Fig 1, right-hand side).

Systems epigenetics is free of the circular logics that plague molecular epigenetics. But its adherents tacitly suppress a fundamental question: if the GRN naturally accounts for the persistence of distinct gene expression configurations, why are local epigenetic modifications necessary in the first place? Merely for the structural function of compacting DNA?

There is a reason why bidirectional reversible modification of chromatin by epigenetic marks cannot produce memory. In engineering, a memory switch must, by definition, be capable of staying ON after a transient signal that turned it ON has subsided, otherwise it is not memory (Fig 1A). The mathematical prerequisite for such ON-OFF (bistable) switching with irreversible dynamics is a circuitry that must contain at least 1 positive feedback loop (plus nonlinearity in its operation characteristics) [8]. The paradigm of systems epigenetics has no difficulty in meeting this requirement because of the plethora of positive feedback loops in the *trans*-acting regulation among the genes of the GRN (Fig 1C).

Two new lines of research from both schools are offering what could be a step towards a logical unification (if they can be generalized to other epigenetic modifier systems). Studies in molecular epigenetics have identified feedback loops capable of bistable switching that satisfies the definition of memory. Holoch and colleagues [9] have demonstrated in a series of elegant experiments that Polycomb complex PRC2 that writes the repressive histone methylation mark H3K27me to silence a target gene X, is itself suppressed by ongoing transcription of X (Fig 1B). Such negation of a negative regulator (PRC2) by the entity being regulated (gene X) constitutes a positive feedback-loop, thus meeting the mathematical requirement for bistability for producing a stable response that outlasts the input signal. A similar feedback-loop has been observed for the c-Kit gene, where high transcription prevents Polycomb from binding to the promoter [10].

But such “*cis*-acting epigenetic memory switches” operate within 1 gene locus and are agnostic of *trans*-regulation between loci. In the systems epigenetics view, they seem unnecessary and certainly not sufficient for establishing and remembering entire expression patterns. Why then have cells evolved these elaborate *cis*-acting bistable switches [9]? What does systems epigenetics miss that give *cis*-acting switches their raison d’être?

A new study has exposed a decoupling of chromatin (de)compaction from the erection of specific transcriptomes, confirming that chromatin remodeling is not the primum movens in coordinating gene expression. Parmentier and colleagues [11] observe that, similar to the early embryo, CD34^+^ blood progenitor cells with multi-lineage potential undergo a global chromatin opening before differentiating into specific lineages. How do lineage-specific expression patterns reliably arise from such “indifferent” access to most genes? Within a few hours of differentiation initiation, 50% of promoters in the genome became accessible, with substantial cell-to-cell variability of the accessible sites, followed by a wave of global stochastic transcriptional bursts [11], consistent with multi-lineage priming of multipotent cells [12]. This genome-wide transcriptional noise was then resolved within 48 h as stable lineage-specific expression patterns emerged.

Computational analysis of single-cell transcriptomes revealed a “critical transition” (tipping-point) dynamic [13] during the stochastic phase, indicating an irreversible transition into a new attractor (a valley on Waddington’s “epigenetic landscape”), which implements the stable lineage-specific transcriptome “intended” by the GRN and shared by all the cells.

One can now hypothesize that the *cis*-acting bistable switches at individual gene loci act like digital transistor switches in computer chips. Compared with analog architectures, digital encoding increases the speed of information processing and provides resistance to noise. Such encoding would accelerate the implementation of specific gene expression “programs” by the GRN. This hypothesis could be tested if the studies of Holoch and colleagues [9] and Parmentier and colleagues [11], each of which subscribe to 1 of the 2 schools of epigenetics, were extended to experiments guided by the respective alternative thought habits.

Thus, the *cis*-acting memory switches (molecular epigenetics) in theory strongly facilitates the implementation of *trans*-regulatory activities hard-wired in the GRN (systems epigenetics). In the formalism of the latter, this would correspond to deepening of the valleys in Waddington’s landscape [3], which increases the stability of genome-wide expression patterns. The 2 disparate schools of epigenetics may represent different facets of the same principle of gene regulation.
